# Efficacy and safety of umbilical therapy with the traditional Chinese medicine formulation *Lishui Xiaogu* cataplasm for cirrhotic ascites: protocol for a randomized controlled trial

**DOI:** 10.1186/s13063-019-3221-y

**Published:** 2019-02-13

**Authors:** Xianzhao Yang, Shuying Ru, Lin Luo, Xiaoying Lv, Wenjing Bai, Fuwen Zhang, Feng Jiang

**Affiliations:** 1 BUCM Institute of Liver Diseases, Dongzhimen Hospital affiliated to Beijing University of Chinese Medicine, No. 5, Haiyuncang, Dongcheng District, Beijing, 100700 China; 2 Department of Gastroenterology, Dongzhimen Hospital Eastern Affiliated to Beijing University of Chinese Medicine, Beijing, 101100 China; 3 Institute of Basic Research in Clinical Medicine, China Academy of Chinese Medical Sciences, Beijing, 100700 China

**Keywords:** Cirrhotic ascites, *Lishui Xiaogu* cataplasm, Traditional Chinese medicine, Umbilical therapy, Randomized controlled trial

## Abstract

**Background:**

Ascites is one of the most common complications of cirrhosis. Umbilical therapy with traditional Chinese medicines has been increasingly prescribed to treat cirrhotic ascites. However, high-quality evidence from clinical trials supporting such application of traditional Chinese medicines remains limited. Therefore, we designed a clinical trial to evaluate the efficacy and safety of umbilical therapy with the *Lishui Xiaogu* cataplasm formulation applied to treat cirrhotic ascites.

**Methods/design:**

This ongoing study is a double-blind, randomized, parallel, placebo-controlled trial. A total of 82 patients will be recruited and randomly assigned to either a treatment group or a placebo group, in a 1:1 ratio. The treatment group will receive umbilical therapy with the *Lishui Xiaogu* cataplasm plus red light irradiation along with conventional treatment; the placebo group will receive umbilical therapy with a placebo cataplasm plus red light irradiation along with conventional treatment. Interventions for both groups will be administered once daily for up to 10 days, with a 30-day follow-up after the last treatment. The primary efficacy measurement will be ascites depth. Secondary efficacy measurements will include abdominal perimeter, weight, urine volume, the symptomatic score of traditional Chinese medicine, and the Chronic Liver Disease Questionnaire. Adverse events will also be reported.

**Discussion:**

This randomized trial will be the first rigorous study designed to evaluate the efficacy and safety of umbilical therapy with *Lishui Xiaogu* cataplasm applied for cirrhotic ascites.

**Trial registration:**

Chinese Clinical Trial Registry, ChiCTR-INR-16007686. Registered on 1 January 2016.

**Electronic supplementary material:**

The online version of this article (10.1186/s13063-019-3221-y) contains supplementary material, which is available to authorized users.

## Background

Liver cirrhosis is a diffuse hepatic process, characterized by fibrosis and structurally abnormal nodules, representing the final histological change due to various chronic liver diseases [1]. Ascites is the most common complication of decompensated cirrhosis, which is often associated with a poor prognosis and impaired quality of life. Mortality among patients with cirrhotic ascites is approximately 40% at 1 year and 44–85% at 5 years [2, 3]. In the absence of proper treatment, large ascites may lead to dyspnea, umbilical hernia, acid–base disturbances, hepatic encephalopathy, hepatorenal syndrome, and even death.

In recent years, a series of clinical practice guidelines regarding the management of ascites has been published [4–6], providing recommendations for ascites treatment including diuretic therapy, large-volume paracentesis, and albumin infusion. However, some limitations remain, particularly concerning the side-effects of the standard treatment for refractory ascites. Therefore, it is crucial to find better practices to successfully manage ascites.

Traditional Chinese medicine (TCM), including internal and external therapeutic approaches, has been used to treat ascites for over 1000 years. According to the meridian–collateral and visceral theories of TCM, the application of herbal remedies to the umbilical region, covered and fixed with a medical gauze and adhesive tapes, along with hot ironing, can play an important role in the treatment of various diseases, including cirrhotic ascites [7]. Previous studies have reported the use of umbilical therapy to treat ascites [8, 9]. However, they lacked a rigorous trial design. Moreover, a previous study performed by our group has demonstrated a significant effect of *Lishui Xiaogu* (LSXG) cataplasm treatment for ascites. Therefore, we designed the present randomized controlled trial (RCT), which obtained special support from the Beijing Capital Characteristic Clinical Application Research Project by the Beijing Municipal Science & Technology Commission of China. Our aim is to evaluate the efficacy and safety of the LSXG cataplasm umbilical therapy in patients with cirrhotic ascites, using a rigorous prospective randomized controlled clinical trial. The findings of this trial may establish the corresponding operational criteria and obtain evidence-based medical evidence, so as to provide an effective alternative measure for the treatment of cirrhosis ascites.

## Methods/design

### Study design and setting

**Fig. 1:**
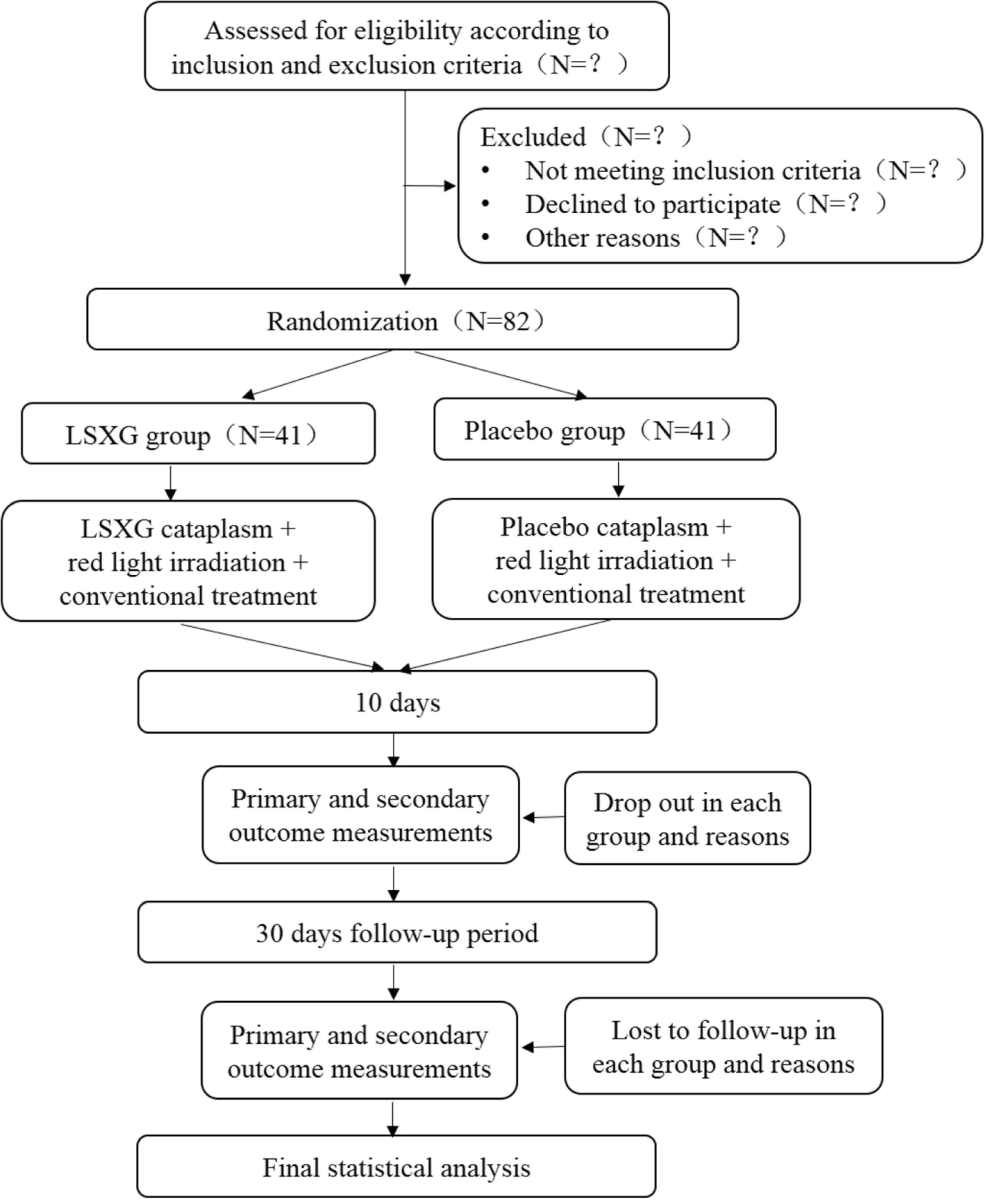
Flowchart of the study design. LSXG *Lishui Xiaogu*

**Fig. 2:**
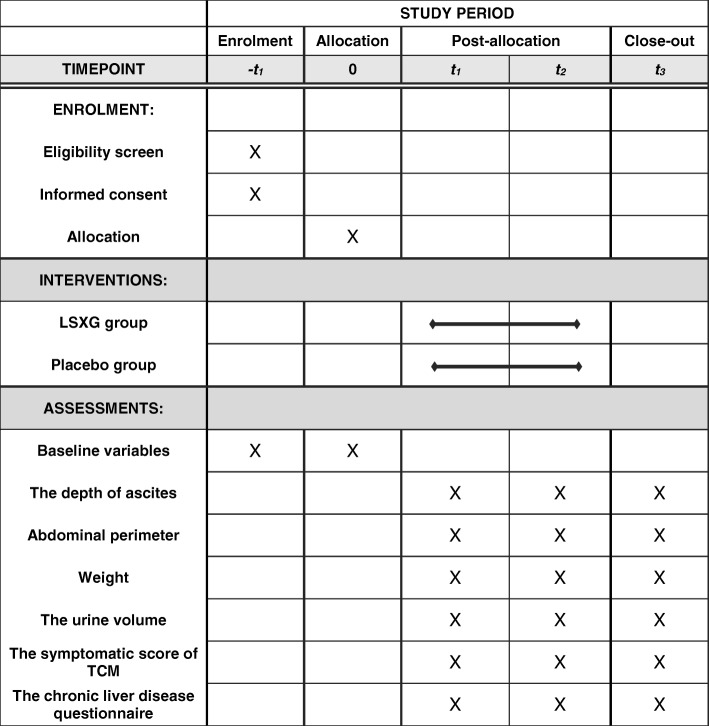
The schedule of enrolment, interventions, and assessments. LSXG *Lishui Xiaogu*, TCM traditional Chinese medicine

This ongoing study was designed as a central-randomized, double-blind, parallel, placebo-controlled, multicenter trial. The study was designed using the Consolidated Standards of Reporting Trials (CONSORT) 2010 guidelines [10]. Also, we designed the trial based on the Standard Protocol Items: Recommendations for Interventional Trials (SPIRIT) statement. The study is being performed in three centers in China, namely: Dongzhimen Hospital, affiliated to Beijing University of Chinese Medicine; Beijing Tongzhou District Hospital of Traditional Chinese Medicine; and Beijing Pinggu Hospital of Traditional Chinese Medicine. Patients with cirrhotic ascites, who meet the inclusion criteria, will be allocated in a 1:1 ratio to either a LSXG group or a placebo group. Patients in the LSXG group will receive umbilical therapy with the LSXG cataplasm plus red light irradiation along with conventional treatment; patients in the placebo group will receive umbilical therapy with a placebo cataplasm plus red light irradiation along with conventional treatment. Based on clinical experience, the treatment will be administered once daily for up to 10 days, with a 30-day follow-up after the last treatment. The depth of ascites will be assessed as the primary outcome measurement. The abdominal perimeter, body weight, urine volume, the symptomatic score of TCM, and the Chronic Liver Disease Questionnaire (CLDQ) will be used as secondary outcome measurements. All assessments will be conducted at three time points: at baseline, after the last treatment, and after the 30-day follow-up period. The flow chart is shown in Fig. 1. We also provide the Standard Protocol Items: A Recommendations for Interventional Trials (SPIRIT) figure (Fig. 2) and the corresponding checklist (Additional file 1).

### Ethics

The study protocol has been approved by the Institutional Review Board of Dongzhimen Hospital, affiliated to the Beijing University of Chinese Medicine (No. ECPJ-BDY-2015-69), and has been registered with the Chinese Clinical Trial Registry (ChiCTR-INR-16007686). The Institutional Review Board will be responsible for supervising all procedures of the study, including patient recruitment, randomization, treatment administration, data storage, and so on. In the case of any changes to the original study protocol, a written application will be submitted to the Institutional Review Board for approval. The study will be conducted according to the Good Clinical Practice Guidelines and the principles of the Declaration of Helsinki. Results will be reported according to the same guidelines.

### Randomization and allocation concealment

The randomization sequence will be generated using SAS software, version 9.4 (SAS Institute). The central randomization system used in this trial was provided by the Institute of Basic Research in Clinical Medicine, China Academy of Chinese Medical Sciences. It is an online platform. Each center staff member logs into the website when the participant is enrolled, enters the basic information of the participant, and then obtains a random number. Eligible participants will be randomly assigned to the LSXG or the placebo group in a ratio of 1:1. All eligible participants will obtain the corresponding umbilical drug according to their random number, and they will be individually treated to prevent communication. The participants, researchers, and statisticians will be blinded to treatment allocation.

### Informed consent

Prior to the study, the general study process and the responsibilities of the participants and researchers will be explained to potential participants. Participants will be informed that their entry into the trial is entirely voluntary and that they can withdraw at any time. In the event of their withdrawal, data collected on the participant will not be erased and will be used in the final analyses. Written informed consent will be obtained from each participant before they undergo any interventions related to the study.

### Inclusion criteria

Participants who meet the following criteria will be included in the study:

1. Presence of grade 2–3 ascites according to the diagnostic standards of liver cirrhosis [5]: cirrhosis patients with symmetrical distension of the abdomen, and ascites depth > 3 cm.
2. Age between 18 and 75 years, irrespective of sex.
3. An agreement to voluntary participation in the trial and the provision of informed consent.

### Exclusion criteria

Participants who meet any of the following criteria will be excluded:

1. Presence of liver cirrhosis decompensation accompanied by bleeding, gastrointestinal hemorrhage, hepatic encephalopathy, hepatorenal syndrome, and other serious complications.
2. Presence of a combination of severe cardiovascular, lung, kidney, neuronal, and blood circulation system diseases as well as other serious illnesses, such as mental illness, or tumors.
3. Current or planned pregnancy, or nursing mothers.
4. Presence of allergic constitution, or a past medical history of allergies to umbilical drugs or their known components.
5. Participation in other clinical trials within 1 month before our study.

### Exclusion criteria

#### Rejection

Participants who meet any of the following exclusion criteria will be rejected:

1. Not following the inclusion criteria.
2. Dropout during the observation period, without any data recorded.
3. Discontinuation of treatment following the baseline time.

#### Discontinuation

Participants who meet any of the following exclusion criteria will be discontinued:

1. Discontinuation of treatment.
2. Occurrence of serious adverse events (AEs) related to the research medication.
3. Occurrence of serious complications, such as gastrointestinal hemorrhage, spontaneous peritoneal inflammation, primary hepatic carcinoma, hepatic encephalopathy, and hepatorenal syndrome.
4. Worsening of disease, which will require emergent treatment.
5. Termination of the trial by the administrative authorities.

Reasons for discontinuation will be recorded on case report forms (CRFs), and the last data recorded for such participants will be included in the data analysis.

#### Dropout

Participants can voluntarily withdraw at any time during the trial. Participants who meet the inclusion criteria, provide a completed and signed consent form, and successfully enter the randomized trial but fail to complete the proposed observational period, regardless of time and reason, will be regarded as dropout cases. Dropout cases include:

1. Poor treatment compliance (< 80%), use of forbidden medicines, or change in treatment without consent from the study supervisor.
2. Voluntary withdrawal or incomplete clinical data, affecting the evaluation of efficacy and safety.
3. Occurrence of serious AEs or complications, resulting in the discontinuation and unblinding of the treatment.

### Interventions

Patients in the LSXG group will receive the umbilical therapy with LSXG cataplasm plus red light irradiation along with conventional treatment, whereas patients in the placebo group will receive the placebo cataplasm plus red light irradiation along with conventional treatment. The treatment will be administered once daily for up to 10 days, with a 30-day follow-up after the last treatment.

#### Traditional Chinese medicine intervention

The LSXG cataplasm comprises Pharbitidis Semen (Qianniuzi) 15 g, Plantaginis Semen (Cheqianzi) 9 g, *Rheum officinale* (Dahuang) 9 g, Coicis Semen (Yiyiren) 9 g, Costustoot (Muxiang) 6 g, and Kansui Radix (Gansui) 6 g. The composition of specific Chinese herbal remedies and their actions are summarized in Table 1 [11]. Placebo cataplasm comprises 1% of the formulation mentioned and 99% dextrin and caramel. It resembles the LSXG formula in appearance, color, and scent. Both cataplasms are manufactured and quality controlled by Beijing Tcmages Pharmaceutical Co., Ltd. (Beijing, China) according to industry standards. All of the drugs are uniformly packaged and identified with the same labels.

**Table 1:** Composition and effects of herbs in the *Lishui Xiaogu* cataplasm

Ingredient	Pharmacological effects according to traditional Chinese medicine
Qianniuzi	Purging water and relieving constipation, clearing away phlegm and expelling water, killing worms, and resolving accumulation
Cheqianzi	Clearing heat, diuresis and treating stranguria, dispelling dampness to stop diarrhea, improving vision, and dispelling phlegm
Dahuang	Contributing to defecation to resolve accumulation, clearing heat and purging fire, cooling blood to remove toxins, removing blood stasis and promoting menstruation, dispersing dampness, and eliminating jaundice
Yiyiren	Eliminating dampness and disinhibiting water, strengthening the spleen to stop diarrhea, removing obstruction, expelling pus, detoxicating, and resolving stasis
Muxiang	Regulating qi to stop pain, and invigorating the spleen to promote digestion
Gansui	Removing retained water and morbid fluid, subduing swelling, and dissipating bind

Administration method: 0.5 g of the formula will be combined with rice wine to form a paste, which will be applied to the patient’s umbilical region for 120 min and then exposed to red light for 20 min once a day.

For cataplasm application: the umbilical region should be thoroughly cleaned with an iodophor solution; the patients must be rested and abstain from stimulating food, such as pungent food, seafood, or raw and cold food, and the cataplasm should not be applied immediately before a meal; the amount of formula administered and the time of administration should be adjusted for weaker patients (any changes in patient conditions should be recorded); if the cataplasm application triggers erythema, blisters, or itching, it should be removed; and the cataplasm should be contraindicated in patients with known allergies to any of the constituents, pregnant patients, and patients presenting with edema with Yin-shui syndrome. This application should be used with caution in weaker patients.

#### Conventional treatment

Following the available clinical guidelines [12, 13], the patients will be provided with conventional general treatment, including rest, limited salt intake (approximately 60–90 mmol of sodium per day), and limited water intake, and etiological treatment, including diuretics (spironolactone (40–100 mg/day) and furosemide (20–40 mg/day), at a ratio of 5:2), liver protection treatment (including reduced glutathione, polyene phosphatidyl choline, tiopronin, silibinin, etc.), and albumin infusion for patients with serum albumin levels of < 25 g/dl.

### Sample size

In our previous study, the estimated total effective size of the study population was 87.5% for TCM application and 62.5% for the control treatment [14]. At *α* = 0.05 and *β* = 0.20, and assuming a dropout rate of 10%, the one-tailed test provided an estimated sample size of 41 patients per group.

### Basic characteristic variables

Collected baseline data include the following:

1. Demographic characteristics, such as sex, age, ethnicity.
2. Biochemical and clinical measurements, such as routine blood, stool, and urine tests; tests for hepatic and renal functions; ion tests; electrocardiography (ECG); and measurement of ascites depth, abdominal perimeter, body weight, and urine output.
3. Main symptoms, such as abdominal distension, appetite, level of exhaustion, and normal/abnormal defecation.
4. Health-related quality-of-life scale for patients with chronic liver disease.
5. Disease course, combined drugs, and other relevant information.

Vital signs, including blood pressure, pulse, respiratory rate, and body temperature, will also be recorded every day.

### Primary outcomes

The primary outcome measurement will be the depth of ascites. It will be measured at baseline using B-ultrasound, after the intervention (10 days), and at the end of the 30-day follow-up period.

### Secondary outcomes

Secondary outcome measurements include the abdominal perimeter, body weight, the 24-h urine volume, the symptomatic score of TCM, and the CLDQ, which will be measured at baseline, after the intervention (10 days), and at the end of the 30-day follow-up period. The abdominal perimeter, weight, and 24-h urine volume will be measured every day from the baseline to the end of the 30-day follow-up period. The symptomatic score of TCM contains the patient’s primary symptoms, such as abdominal distension, appetite, level of exhaustion, normal/abnormal defecation, and so on. The CLDQ is a disease-specific tool designed to assess health-related quality of life in patients with chronic liver disease [15].

### Safety assessment

Primary vital signs, physical examinations, and some laboratory tests will be performed both before and after treatment for safety assessment daily. Primary vital signs include body temperature, blood pressure, heart rate, and respiratory rate. Laboratory tests include routine blood, urine and stool tests, along with fecal occult blood tests; ECG; liver function tests (alanine aminotransferase (ALT), aspartate aminotransferase (AST), alkaline phosphatase (ALP), gamma-glutamyl transpeptidase (GGT), and serum total bilirubin (TBIL)); renal function test (serum creatinine (Cr) and blood urine nitrogen (BUN)); and electrolyte test (K^+^, Na^+^, Cl^−^, and Ca^2+^).

### Adverse events

All unexpected AEs occurring during the intervention period will be reported, and the causality related to the LSXG cataplasm will be analyzed. If any adverse event occurs, the study managers will ensure that the participant receives adequate treatment. The AEs will be immediately reported to the principal investigator and to the ethics committee to decide whether the participant should withdraw from the trial. If any symptoms aggravate, the patient will be withdrawn from the study and will be referred for further treatment.

### Statistical analysis

The data from all participating centers will be combined for statistical analysis of the primary and secondary outcomes as well as the AEs. The statistical analysis will be performed at the Institute of Basic Research in Clinical Medicine, China Academy of Chinese Medical Sciences. The primary outcome will be analyzed according to the intention-to-treat principle. To compare the difference between two groups, independent *t* tests (or the Mann–Whitney *U* test) will be used to analyze continuous data and the chi-squared or Fisher’s exact test will be used for categorical data. Repeated-measures ANOVA will be used to account for the time effect.

For all measures, two-sided *p* < 0.05 will be considered statistically significant. Statistical analyses will be performed using SAS software version 9.4 (SAS Institute, Cary, NC, USA).

## Discussion

Umbilical therapy with TCM formulations has a long history in China, and has been popularly used to treat various diseases, including cirrhotic ascites. Through being applied to the umbilical region, TCM may be absorbed into the blood quickly and stimulate peripheral nerves, which may be the reason for its action. Also, as a complementary therapy, it is easy to operate and convenient, compared with acupuncture, moxibustion, and TCM soups.

Previous studies preliminarily showed that umbilical therapy is effective and safe in treating cirrhotic ascites [8, 9, 16–19]. One meta-analysis demonstrated that umbilical therapy was more effective in increasing 24-h urinary volume, decreasing patients’ weight and abdominal circumference, compared with conventional medicines. Adverse reactions were rarely reported, which often include skin itching or partial flushing due to the adhesive plaster [8]. Another meta-analysis of mirabilite umbilical compress in treatment of ascites in liver cirrhosis showed similar results [9]. Application of Shehuang Paste to the umbilical area was also reported to reduce blood flow of the portal vein and the splenic vein, and to lower the content of endotoxin, nitric oxide, and endothelin-1 [8].

However, the lack of high-quality evidences limits the application of umbilical therapy [8]. Most studies were small sample, unblind, and based on one medical center, which may lead to bias caused by confounding factors. Besides, some studies took the 24-h urinary volume, patients’ weight, and abdominal circumference change as primary outcomes, which could not reflect the dynamic change of ascites directly [18, 20]. Based on previous research [14], our study will be the first central-randomized, double-blind, parallel, placebo-controlled, multicenter trial, using the dynamic change of ascites by B-ultrasound as the primary outcome. In conclusion, we present the protocol of a randomized controlled, double-blind, multicenter trial, aiming at evaluating the efficacy and safety of umbilical therapy for cirrhotic ascites, as a complementary and alternative therapy. Results of the current study will provide high-quality evidence supporting the management of ascites using umbilical therapy with TCM.

## Trial status

The trial has been approved and registered. At the time of manuscript submission, the study has been actively enrolling subjects and, as of this writing, has a total of 78 subjects. The study is ongoing.

## Additional file

Additional file 1: SPIRIT 2013 checklist. (DOC 124 kb)

## Abbreviations

AE: Adverse event
ALT: Alanine aminotransferase
AST: Aspartate aminotransferase
CLDQ: Chronic Liver Disease Questionnaire
CONSORT: Consolidated Standards of Reporting Trials
CRF: Case report form
ECG: Electrocardiography
LSXG: *Lishui Xiaogu*
RCT: Randomized controlled trial
SAS: Statistical Analysis System
SPIRIT: Standard Protocol Items: Recommendations for Interventional Trials
TCM: Traditional Chinese medicine
GGT: Gamma-glutamyl transpeptidase
